# Risk assessment for the implementation of controlled human *Schistosoma mansoni* infection trials in Uganda

**DOI:** 10.12688/aasopenres.12972.2

**Published:** 2019-08-13

**Authors:** Jan Pieter Koopman, Moses Egesa, Anne Wajja, Moses Adriko, Jacent Nassuuna, Gyaviira Nkurunungi, Emmanuella Driciru, Gijsbert van Willigen, Stephen Cose, Maria Yazdanbakhsh, Pontiano Kaleebu, Narcis Kabatereine, Edridah Tukahebwa, Meta Roestenberg, Alison M. Elliott

**Affiliations:** 1 Department of Parasitology, Leiden University Medical Center, Leiden, The Netherlands; 2 Uganda Virus Research Institute, Entebbe, Uganda; 3 Medical Research Council/Uganda Virus Research Institute and London School of Hygiene & Tropical Medicine Uganda Research Unit, Entebbe, Uganda; 4 Vector Control Division, Ministry of Health of Uganda, Kampala, Uganda; 5 Department of Health, Safety and the Environment, Leiden University Medical Center, Leiden, The Netherlands; 6 Clinical Research Department, London School of Hygiene & Tropical Medicine, London, UK

**Keywords:** Schistosoma mansoni, Controlled Human Infection Studies, Uganda, risk assessment

## Abstract

Schistosomiasis is a parasitic infection highly prevalent in sub-Saharan Africa, and a significant cause of morbidity; it is a priority for vaccine development. A controlled human infection model for *Schistosoma mansoni* (CHI-S) with potential to accelerate vaccine development has been developed among naïve volunteers in the Netherlands. Because responses both to infections and candidate vaccines are likely to differ between endemic and non-endemic settings, we propose to establish a CHI-S in Uganda where *Schistosoma mansoni *is endemic. As part of a “road-map” to this goal, we have undertaken a risk assessment. We identified risks related to importing of laboratory vector snails and schistosome strains from the Netherlands to Uganda; exposure to natural infection in endemic settings concurrently with CHI-S studies, and unfamiliarity of the community with the nature, risks and rationale for CHI. Mitigating strategies are proposed. With careful implementation of the latter, we believe that CHI-S can be implemented safely in Uganda. Our reflections are presented here to promote feedback and discussion.

## Background

Schistosomiasis is a parasitic infection affecting approximately 230 million people worldwide ^1^. Infection is caused by trematodes (flukes) of the genus *Schistosoma.* Because the infection is responsible for considerable morbidity worldwide, particularly in Africa, schistosomiasis was recently listed among the top 10 infections for which a vaccine should urgently be developed ^2^.

Controlled human infection (CHI) studies are an important tool for vaccine development. They provide a platform to safely and swiftly test vaccine candidates for the pathogen in question. Furthermore, they can contribute to understanding host-pathogen interactions and help to unravel the nature of protective immunity. They have been used successfully for a substantial number of infectious diseases, including malaria, dengue, and influenza ^3^. A CHI model has now been developed for schistosomiasis at Leiden University Medical Center, where Dutch volunteers with no previous exposure to schistosomiasis participated ^3^. However, the response to schistosome infection, and to candidate vaccines, is likely to be different in endemic countries. In such settings multiple differences in environmental exposures, as well as prior exposure to schistosomes, drive differences in both the innate and adaptive immune responses which determine infection susceptibility and vaccine responses ^4, 5^.

We are therefore working towards the establishment of a controlled human infection model for schistosomiasis in Uganda, where *Schistosoma mansoni* is highly endemic. Almost 30% of the population is estimated to be infected ^6^, with half the population at risk ^7^. As a first step we held a stakeholders’ meeting in Uganda in November 2017, and we published the meeting report and resultant road-map for the implementation process ^8^. A key element of the road-map was to undertake a risk assessment. This document therefore aims to provide an assessment of risks that may arise before, during and after start of a controlled human infection model with *Schistosoma mansoni* (CHI-S) in Uganda.

Male and female schistosomes live in the mesenteric or perivesical veins of their human host, where they mate and produce eggs. These eggs are either released into the environment through faeces and urine or stay within the host tissue where they induce inflammation. When the excreted eggs reach fresh water, they hatch and release miracidia that can then infect a suitable snail host. Infected snails are able to shed larvae, called cercariae, which infect humans. The Leiden University Medical Center (LUMC) CHI-S exposed healthy naïve volunteers to increasing doses of male cercariae to study the tolerability of such a controlled human infection model. This male-only model avoids the risk of pathology caused by schistosome eggs. To generate the infectious cercariae for a male-only CHI-S, individual laboratory-reared freshwater snails are infected, each with a single miracidium. Clonal replication follows, such that thousands of single-sex cercariae are subsequently shed by the snail. The sex of the cercariae can be determined by PCR, and the appropriate number of cercariae can be prepared for dermal infection. Because snails shed thousands of cercariae over a period of weeks, every time they are exposed to light, it is possible to first perform quality control (QC) testing on every batch (e.g. to assess the viability, sex and bioburden of the cercariae). Following principles set forward in good manufacturing practices (GMP) guidelines, the cercariae and their excipients are produced and tested for consistent quality according to predefined criteria. Only when compliant, is the cercariae batch released for clinical use. To this date, 17 people have been exposed to *S. mansoni* cercariae during CHI-S studies in Leiden.

In terms of the technical aspects of shipping infectious material to Uganda, culturing the infectious material in Uganda and preparing the infectious cercariae, we have considered three options. 

**Option 1: Shipping of parasites and snails from the Netherlands to Uganda.** In this scenario, *S. mansoni* parasites and snails would be shipped from Leiden (The Netherlands) for preparation of the cercariae for human infection in Uganda. From a technical perspective, the easiest approach to rapid implementation of CHI-S in Uganda would be to produce and release the infectious snails in Leiden and subsequently ship them to Uganda. In Uganda, a further snail shedding would be used to generate the infectious cercariae. Alternatively, *S. mansoni* parasites (for example in the form of *S. mansoni* eggs contained in a rodent liver) could be shipped separately from uninfected snails, which would mitigate shipment risks.

The CHI-S model in Leiden uses a schistosome strain which has been genotyped and has been mapped to be of Puerto Rican origin ^3^. Because this strain has been laboratory adapted and kept in the Leiden facility since 1955, it has the advantage of its known virulence in animals, experience of its effects in the Dutch human volunteers, and its sensitivity to praziquantel. As well, the Leiden model uses *Biomphalaria glabrata* snails which are not indigenous to Uganda (Appendix 1 [Extended data ^9^]). Therefore, the ecological risks of accidental release of schistosomes or snails or into the environment have to be considered.

**Option 2: Shipping of parasites from The Netherlands followed by use of local Ugandan snails.** This scenario would involve transporting only *S. mansoni* parasites (Puerto-Rican strain), then using local snail species such as *B. choanomphala* (from Lake Victoria) or *B. stanleyi* (from Lake Albert) to produce cercariae in Uganda ^10^. Advantages, as in option 1, would be the fact that the parasite strain has been characterized in both animals and humans, which decreases its potential risk for the volunteers. Disadvantages would be possible technical hurdles to be overcome to establish a local snail colony and achieve successful infection with release of infectious *S. mansoni* cercariae. However, expertise in these processes already exists in Uganda ^10^, subject to laboratory renovations and staff training to ensure compliance with GMP principles. This option would also be relatively simple to implement.

**Option 3: Using local Ugandan parasites and local Ugandan snails.** In this scenario the full *S. mansoni* laboratory life cycle would be established in Uganda, using a local snail species and starting with a new *S. mansoni* strain, and a rodent mammalian host. Although the risk of clinically unexpected, unwanted side effects, or of relative resistance to praziquantel treatment, might be higher when using the local strain of *S. mansoni*, the ecological risk would be lowest.

All options require preparation of the cercariae for human infection under strict Quality Assurance and controlled conditions in Uganda with adherence to Good Manufacturing Guidelines. In Leiden, procedures were developed based on GMP principles contained in the European Commission directive 2003/94/ EC, with the infectious cercariae considered as an “auxiliary medicinal product”. Details of the procedures have been published ^3^. These include production in a biosafety level 3 facility, governed by stringent standard operating procedures including for quality control, logging and monitoring; production and counting of infectious cercariae by two independent technologists; and antibiotic treatment and microbiological bioburden testing to ensure that the cercarial product is free of pathogens with potential to harm CHI volunteers. Equivalent procedures and quality control will be needed in Uganda in order to implement CHI-S.

In this document we address risks associated with CHI-S in Uganda on three different levels: i) the introduction of new species (the transport of snails, the snail culture facilities, the potential for ecological harm as a result of importing snails), ii) the introduction of a new schistosome strain into Uganda, and iii) clinical trial risks common to all options (natural infection during the trial period, and the risks to volunteers resulting from the controlled infection).

## Risk assessment methods

We identified risks and potential approaches to mitigation based on relevant literature, experience from the Leiden CHI-S model, stakeholder discussions, and discussion with experts. The level of risk and effectiveness of proposed controls was determined by consensus between the authors. The inherent risk was defined as the risk before putting controls in place, calculated as the product of the likelihood and impact scores. The residual risk was similarly calculated, based on likelihood and impact scores after controls have been put in place. Mitigating controls could reduce the residual risk score by reducing the likelihood of an event occurring, or by reducing the impact if it should occur. Likelihood was scored as almost certain/common, 5; likely, 4; possible, 3; unlikely, 2; rare, 1. Impact was scored as critical, 5; major, 4; moderate, 3; minor, 2; insignificant 1. Resulting risk scores of 18–25 were considered high, and unacceptable. Resulting risk scores in the range of 9–17 were considered moderate, with further controls desirable if possible, and caution required if implemented at this risk level. Resulting scores of 0–8 were considered low, and usually acceptable.

## Option 1: Shipping of parasites and snails from the Netherlands to Uganda

According to our first idea, infected snails would be shipped. The WHO report ‘Guidance on regulations for the Transport of Infectious Substances 2017–2018’ ^11^ provides information on how to adequately transport infectious substances. In accordance with these guidelines, shipment of *S. mansoni* infected snails falls under ‘CATEGORY B, INFECTIOUS SUBSTANCES’ (UN3373). Shipment of live snails is a time-sensitive undertaking and therefore can only be facilitated by air shipment. Infectious substances cannot be carried on as hand-luggage. Transport of infectious substances are subjected to International Air Transport Association (IATA) requirements. Packaging of Category B substances need to comply with rules set out in the P650 packaging instruction ^11^. This involves triple packaging and proper marking and documentation. Upon arrival in Uganda, it would be crucial for the package to clear customs as quickly as possible so that snails arrive in good condition. In order to achieve this, the customs office should be notified about the arrival of the shipment. In collaboration with the customs officer, all required documentation should be prepared in advance and approval for import of the products should be sought.

Alternatively, snails and *Schistosoma* parasites would be shipped separately. Uninfected snails can be shipped more easily because this shipment does not have to comply with the regulations for the transport of infectious substances. Similar to the previous option, shipment should clear customs as soon as possible. These snails could be kept to reproduce in the Ugandan laboratory to sustain their life cycle.

A second shipment would contain *Schistosoma* parasites. There are two ways in which this material can be transported (still under the ‘CATEGORY B, INFECTIOUS SUBSTANCES’ (UN3373)):

1) Within a living host such as a Schistosoma-infected hamster. These animals can shed *Schistosoma* eggs that can be used to infect the snails.

2) Within a preserved liver sample kept on medium from a *Schistosoma*-infected hamster. This liver sample contains *Schistosoma* eggs. Upon arrival in Uganda, further processing of the sample provides miracidia which can be used to infect the snails. Test shipments should be scheduled to determine the feasibility of such transports and the conditions in which the liver sample should be shipped. From previous experiments in Leiden, the preserved liver sample can be used to infect snails for up to one week after being harvested.

Risks associated with shipping of parasites and snails from the Netherlands to Uganda, and mitigating strategies, are summarized in Table 1.

**Table 1.** Risks associated with shipping of *Schistosoma mansoni* parasites and *Biomphalaria glabrata* snails.

Risk	Inherent risk score		Total inherent risk	Controls	Residual risk score		Total risk post control
	Likelihood	Impact			Likelihood	Impact	
Death of snails in transport	Likely	Critical	20	Pilot transport with low numbers of snails to optimize transport conditions	Possible	Critical	15
Delays in customs clearance	Likely	Major	16	Contacting customs officials to discuss required documentations and preparing documents prior to shipment	Possible	Major	12
Spill of infectious materials and non- indigenous snail species	Possible	Major	12	Proper packaging	Unlikely	Moderate	6
Establishment of a *B. glabrata* colony outside laboratory facility	Possible	Critical	15	Proper packaging	Rare	Critical	5

Likelihood was scored as almost certain/common, 5; likely, 4; possible, 3; unlikely, 2; rare, 1. Impact was scored as critical, 5; major, 4; moderate, 3; minor, 2; insignificant 1.

## Option 1: snail culture facilities; potential ecological harm

To house the *Biomphalaria glabrata* snails in Uganda, they would need to be kept in strict quarantine. *B. glabrata* are not a naturally occurring snail host in Uganda, and should therefore not spread to the environment. In order to house snails, an incubator, or room temperature, set and monitored at 28°C is needed. The incubator (if used) door should be fully closed when the laboratory is not in use. Precautionary measures to contain the snails to the facility should be taken and include physical barriers, such as rooms with closed doors and windows. The snail culture basins and water drainage system should be covered with fine mesh to prevent escape (appendix 1 [Extended data ^9^]). In addition, access to the laboratory should be controlled and restricted to the research team. The incubator (if used) should preferably be positioned away from the door. Additional security measures could be a double door to create a sluice. Appendix 2 (Extended data ^9^) lists precautionary measures that should to be taken when working with schistosomes. Standard operating procedures (SOPs) will be exchanged with LUMC and reviewed to fit the Ugandan facility. These SOPs deal with culture processes as well as the disposal of infectious material.

In case a single snail would accidentally be released into the environment, it is capable of reproducing in the absence of an opposite-sex snail using self-insemination ^12^. This ability poses an ecological hazard where a single snail could develop into a colony. In addition, snails can be transported over large distances attached to birds and can survive dry conditions for up to two months. This snail itself is not endemic in Uganda, although previously this species has been held at the Vector Control Division of the Ministry of Health for a different project. The consequences of accidental introduction of this new species are difficult to predict, however it may result in the following (Appendix 1 [Extended data ^9^]):

1) Interspecific hybridization between *B. glabrata* and local *Biomphalaria* species
2) Uncontrolled spread due to lack of natural enemies, competitors or pathogens
3) Altered *S. mansoni* dynamics, because of potentially higher susceptibility of *B. glabrata* for *S. mansoni* infection

Spread to the environment of *B. glabrata* may go unnoticed, because of its similar morphology to endemic snail species.

Risks associated with culture of *B. glabrata* in Uganda, and mitigating strategies, are summarised in Table 2.

**Table 2.** Risks associated with snail culture facilities.

Risk	Inherent risk score		Total inherent risk	Controls	Residual risk score		Total risk post control
	Likelihood	Impact			Likelihood	Impact	
Spread of *Biomphalaria* *glabrata* snail to environment	Possible	Critical	15	1) Precautionary measures for snail housing facility including physical barriers and restricted access 2) Use of SOPs regarding disposal of infectious material and non- indigenous snail species	Rare	Critical	5
Establishment of a *B. glabrata* colony outside laboratory facility	Possible	Critical	15	1) Development of containment strategies	Rare	Critical	5

Likelihood was scored as almost certain/common, 5; likely, 4; possible, 3; unlikely, 2; rare, 1. Impact was scored as critical, 5; major, 4; moderate, 3; minor, 2; insignificant 1.

## Option 2: transport of *S. mansoni* infectious material and use of local snail species for cercarial production

This approach only requires transport of *S. mansoni* infectious material. This would use the second transport approach described in option 1, within a preserved liver sample from a schistosomiasis-infected hamster. The same regulatory guidelines for transporting infectious material apply. With regard to Ugandan snail species, there is variability between snail species in susceptibility to *S. mansoni* infection; however, there is experience of conducting infection of local species at the Vector Control Division ^10^, so this is expected to be feasible. A major advantage of this approach is that the potential ecological and genetic risks related to introduction of a non-endemic snail species can be avoided.

## Option 3: re-establishing the full *S. mansoni* laboratory life cycle in Uganda, using a local snail species and *S. mansoni* strain

The alternative to shipping infectious material and snails from The Netherlands is to re-establish the full laboratory life cycle of *S. mansoni* using Ugandan snail species and Ugandan isolates of *S. mansoni*. The life-cycle has been maintained in the past at the Vector Control Division of the Ministry of Health, but is not currently available. The advantages of using a Ugandan life cycle include reducing the environmental risk associated with non-endemic snail species and schistosome strains. In addition, this model would be most representative of the field infections in Uganda. Similar to option 2, although susceptibility to *S mansoni* infection varies between snail species, we do not expect this to be an issue, because the Vector Control Division has experience in infecting local species. There are however several challenges with using Ugandan snails and isolates. With regard to the new schistosome laboratory strain, the characteristics of this would be unknown in terms of virulence and susceptibility to praziquantel treatment. Determining these characteristics would not be simple, since validated tests for schistosome resistance are currently not available. In addition, the new isolate would not be clonal and variability within the newly collected schistosome population might result in variable responses in the host, and to drug treatment. An inbred Ugandan strain could be achieved by crossing clonal males and clonal females to produce a single F1 generation and subsequently cloning the offspring through snails followed by another crossing. This procedure would need to be repeated several times to be able to generate a reasonably monomorphic strain. This process would be laborious and time-consuming and might also result in quite atypical parasites, not necessarily representative of the Ugandan population of schistosomes in general. Ugandan populations have been exposed to regular praziquantel treatment for over a decade, so there is a risk that the initial isolates would include individuals with relative praziquantel resistance ^13^ and could not be established with certainty in the initial stages of the above process. Starting with a more diverse selection of cercariae would generate a more representative laboratory population of Ugandan schistosomes, but would mean that the characteristics of any particular clone (notably pathogenicity or praziquantel resistance) selected for CHI-S would be unpredictable.

Options 1, 2 and 3 all require the establishment of facilities in Uganda for production of the infectious cercariae under GMP principles, in order to ensure high quality, reproducible infectious doses. Option 3 requires also the establishment of suitable, specific pathogen free animal facilities to house the rodents (hamsters or mice) that will provide the mammalian hosts in the laboratory life cycle. Risks associated with these elements are also considered here ( Table 3).

**Table 3.** Risks associated with re-establishing Uganda *Schistosoma mansoni* life cycle.

Risk	Inherent risk score		Total inherent risk	Controls	Residual risk score		Total risk post control
	Likelihood	Impact			Likelihood	Impact	
New isolates of *S. mansoni* from the Ugandan population might exhibit variable praziquantel susceptibility, or praziquantel resistance	Possible	Critical	15	1) Test new isolates for praziquantel susceptibility *in vitro* and in an animal model before use in CHI	Unlikely	Critical	10
New isolates of *S. mansoni* from the Ugandan population might exhibit unexpected virulence	Possible	Critical	15	1) Test new isolates for relative virulence in an animal model before use in CHI	Unlikely	Critical	10
Production processes based on GMP principles for single- sex infectious cercariae not established in Uganda	Possible	Critical	15	1) Development of appropriate animal and snail facilities 2) Training of Ugandan staff 3) Monitoring and review by experienced LUMC collaborators 4) Monitoring and review by Ugandan regulators	Rare	Critical	5

Likelihood was scored as almost certain/common, 5; likely, 4; possible, 3; unlikely, 2; rare, 1. Impact was scored as critical, 5; major, 4; moderate, 3; minor, 2; insignificant 1.

## Natural infection during trial period

The single-sex *S. mansoni* challenge has been designed to prevent the occurrence of egg-associated morbidity. In the current model, volunteers participating in the trial will be infected using only male cercariae which penetrate the skin and result in patent infection. In future, a single-sex female cercariae model may also be used to infect volunteers. The sex of the male cercariae can be determined using a specifically designed multiplex real-time PCR which has been described elsewhere ^3^. Once infected, individuals should avoid any exposure to contaminated water. If a subject were to be naturally infected over the course of the study, this might lead to mixed, male and female, infections, with mating of the schistosomes resulting in egg production that causes morbidity. If the Puerto Rican strain used in Leiden is imported for use in Uganda, mating and (if adequate sanitation is not used) excretion of eggs into the environment could alter the genetic make-up of Ugandan schistosome populations, with unknown consequences. However, given the fact that the Puerto Rican strain has been kept in rodents for >60 years, it seems likely that fitness in humans will be, if anything, lower than Ugandan human strains. Moreover, given that the Puerto-Rican strain is relatively inbred after prolonged passage in the laboratory, and was shown to be praziquantel-sensitive in the CHI-S, hybridisation with Ugandan schistosome populations is unlikely to result in increased praziquantel resistance or virulence.

The chance of natural infection can be limited by choosing a study population which does not come into contact with freshwater. However, this would over-restrict recruitment from the true target population, which is people at risk of *S. mansoni* infection. Options to minimise this risk among volunteers from the preferred target population include the following:

1) The feasibility of avoiding fresh water may be surveyed using questionnaires in a pilot study at the field site and the information used to select volunteers least at risk of re-exposure, and to make provisions to support volunteers to avoid re-exposure.
2) While selecting subjects, the investigator may ask whether the subject is likely to spend time in, or to travel to, areas where the risk of contracting a natural infection is high. If so, once again it should be stressed that contact with fresh water should be avoided; volunteers unlikely to achieve this would be excluded.
3) Apart from providing information to the volunteer and raising awareness of this issue, frequent testing for eggs in stool and urine samples may be performed by microscopy (and PCR). Eggs can be found 5–7 weeks after mixed male and female infection ^1^. *S. mansoni* eggs in stool would indicate a concomitant natural infection, which would necessitate immediate treatment of the volunteer with praziquantel. However, stool microscopy and PCR is likely to be unreliable given variable egg excretion and the low sensitivity of stool examination for eggs ^14^.
4) In those trials in which natural infection may be a considerable risk, testing using plasma circulating anodic antigen (CAA) may be conducted weekly from the outset of the trial. Both natural and experimental infections may then be terminated as soon as patent infection has been detected (e.g. at ~7 weeks post controlled human infection, when CAA levels > 1pg/mL). Early abrogation of the infection will prevent mating and egg laying. There would be modest drawbacks to the resulting data, because it would not be possible to study the dynamics of antigen excretion over time and quantitation of infection would be less accurate.
5) Alternatively, volunteers may be displaced to a non-endemic region for the study duration. However, the prolonged, seven to 12-week “admission” required for the CHI-S would be a major burden and inconvenience, as opposed to the relatively short-duration (24 days) for malaria CHI studies where such approach has been employed ^15^. The possibility of volunteers absconding during the study, given the long duration, might be significant, abrogating the value of such an approach. Additionally, this would have cost implications, in terms of providing suitable accommodation and compensation for loss of income.

Risks associated with natural infection during the CHI-S, and mitigating strategies, are summarised in Table 4.

**Table 4.** Risks associated with natural infection during trial period.

Risk	Inherent risk score		Total inherent risk	Controls	Residual risk score		Total risk post control
	Likelihood	Impact			Likelihood	Impact	
Mixed sex infection in trial volunteers	Likely	Moderate *	12	1) Avoidance of fresh water bodies during trial period 2) Pilot survey to establish feasibility of fresh water avoidance 3) Selection of trial volunteers with low risk of contracting natural infection 4) Abrogation of infection as soon as the trial endpoint has been reached (e.g. CAA> 1 pg/mL) 5) Displacement of volunteers to non-endemic setting with excellent water and sanitation facilities	Rare	Moderate	3
Mixed sex infection in trial volunteers leading to release of Puerto Rican strain into environment	Likely	Moderate	12	1) Full clearance of infections before trial starts 2) Continuous screening for egg production 3) Abrogation of infection as soon as the trial endpoint has been reached (e.g. CAA> 1 pg/mL) 4) Displacement of volunteers to non-endemic setting with excellent water and sanitation facilities.	Rare	Moderate	3

Likelihood was scored as almost certain/common, 5; likely, 4; possible, 3; unlikely, 2; rare, 1. Impact was scored as critical, 5; major, 4; moderate, 3; minor, 2; insignificant 1. * The impact of natural co-infection on morbidity is classed as moderate (rather than major or critical) since volunteers who acquire such an infection would presumably be at risk of mixed-sex natural infections as a result of their usual behaviours and occupation. The risk of egg-related morbidity due to the presence of male worms from the CHI-S would therefore add little to the risk resulting from exposure to natural infection. CAA - circulating anodic antigen

## Risks to volunteers resulting from the controlled human infection

Controlled infection with *S. mansoni* has been successfully performed in 17 Dutch volunteers. Although the single sex infection does not cause egg-related morbidity in volunteers, it may cause symptoms in response to the infection. These include dermatitis due to the percutaneous penetration of the cercariae and an acute schistosomiasis as a consequence of a systemic hypersensitivity response ^16^. Severe acute schistosomiasis syndrome (Katayama fever) may present with symptoms such as fever, fatigue, myalgia, malaise, non-productive cough, eosinophilia and patchy infiltrates on chest radiography. In Leiden, several volunteers reported with systemic symptoms which seemed to be an acute schistosomiasis syndrome, with one volunteer presenting with prolonged symptoms of Katayama fever ^16^. In addition, one volunteer presented with peri-orbital oedema which lasted one day, and may have been related to the infection ^16^. Such symptoms can be treated symptomatically and all recovered. Both these volunteers had received the highest dose of cercariae (30 cercariae) used in Leiden. The risk of severe symptoms can be minimised by dose escalation in modest increments. The impact can be reduced by careful monitoring, provision of symptomatic relief and abrogation of infection by treatment if necessary. Frequent follow up visits need to be scheduled throughout the trial to discuss adverse events and conduct clinical assessments of the study volunteers. Safety laboratory tests need to be routinely performed. Volunteers can also experience side effects related to the praziquantel treatment. Common side effects include nausea, dizziness, and fatigue. Volunteers can be reassured that these symptoms are well recognised and transient. Their severity can be reduced by taking praziquantel after food. Symptomatic relief can be provided when required.

The 2017 stakeholders’ meeting identified community engagement to ensure proper understanding of the CHI-S as an essential basis for ethical conduct of a CHI study. CHI is a novel concept in Uganda, where CHI have not been undertaken in the past and understanding of medical research, in general, is at a low level. The idea of a “medical” procedure being undertaken which is expected to cause symptoms, and undertaken for the greater, rather than an individual, good needs careful explanation. Rumours and misunderstandings have the potential to critically affect the work, and to have an adverse effect also on other institutional research activities. Engagement with national and community leaders, work with community advisory boards who can identify, and help to address, misinformation; effective education of volunteers to a full understanding of the expected effects of the CHI (and reasons for undertaking it) will all be essential to the smooth and safe running of these projects. Experiences from the first malaria CHI in Kenya give helpful guidance as to which issues are particularly relevant to participants and may require careful explanation ^17^.

Volunteers will receive remuneration for participating in the trial to reimburse for expenses and compensate for time and burden of participation. Careful consideration will need to be given to determine the exact amount of the remuneration to avoid coercion. Recent remuneration guidelines from Malawi can help to calculate the amount ^18^. In addition, formative research is currently being undertaken to explore within the target community what remuneration would be considered appropriate and acceptable.

Risks related to volunteers and communities during the CHI-S, and mitigating strategies, are summarised in Table 5.

**Table 5.** Risks associated with controlled human infection with *Schistosoma mansoni*.

Risk	Inherent risk score		Total inherent risk	Controls	Residual risk score		Total risk post control
	Likelihood	Impact			Likelihood	Impact	
Symptoms related to infection	Common	Major	20	1) Slow dose escalation in modest increments 2) Frequent follow up visits and collection of adverse events. 3) Clinical assessment and routine safety lab. 4) Symptomatic treatment with corticosteroids or abrogating infection with praziquantel (which kills adult worms) if needed. 5) Abrogate infection with artesunate (which kills immature forms)	Common	Moderate	15
Symptoms related to treatment with praziquantel	Common	Moderate	15	1) Take praziquantel with food 2) Clinical assessment, reassurance, symptomatic relief if needed	Common	Minor	10
Misunderstanding of the nature of CHI-S studies	Likely	Critical	20	1) Education of community leaders, opinion makers and regulators 2) Work with community advisory board 3) Education of potential volunteers using tested materials 4) Informed consent verified with tests of comprehension	Possible	Major	12
Inappropriate remuneration leading to coerced participation	Possible	Moderate	9	1) Formative research to determine appropriate remuneration	Unlikely	Moderate	6

Likelihood was scored as almost certain/common, 5; likely, 4; possible, 3; unlikely, 2; rare, 1. Impact was scored as critical, 5; major, 4; moderate, 3; minor, 2; insignificant 1.

## Discussion

In this document we have reflected on the potential risks involved in establishing a controlled human infection model for schistosomiasis in Uganda. The opinions expressed and risk scores allocated have been arrived at by discussion between the authors and are therefore subjective. In submitting this document to open peer review through the African Academy of Sciences Open Research Platform we welcome discussion of these issues.

Based on the assessments made, our own reflections and proposed plans are as follows.

First, we have decided not to pursue the option of importing *B. glabrata* snails from the Netherlands to Uganda. Although the proposed controls were estimated to reduce the risk or establishing a colony outside the laboratory to low, it seems unnecessary to incur them. Since snail species endemic to Uganda are susceptible to *S. mansoni* infection we expect that option 2 will work. 

Second, we propose to further pursue the option of using the Puerto Rican laboratory strain of *S. mansoni* in the CHI-S in Uganda. We consider that the recognised virulence and praziquantel susceptibility profile of this strain makes it the safest option for CHI-S and have decided to have safety prevail over the ecological risk. The long-term in-breeding of the laboratory strain is an asset in this regard, making the characteristics of each clone of male cercariae reasonably predictable, and the strain possibly less fit as compared to circulating Ugandan strains. We also believe that the ecological risk of possible spread of the Puerto Rican strain of Sm will be minimized with the proposed measures.

To generate infectious cercariae for human infection and challenge studies following the principles of GMP it will be essential to establish a suitably controlled snail facility in Uganda. For sustainability (to avoid the need of repeated shipping of infectious material from the Netherlands) it will also be necessary to establish a specific pathogen free animal facility to house the mammalian host and complete the laboratory life cycle.

With regard to the selection of volunteers, and avoidance of natural infection during the CHI-S, current activities include engagement with relevant Ugandan communities which are potential settings for recruitment of volunteers. As part of the engagement, options for avoidance are being explored. Our current view is that careful volunteer selection, close follow up and immediate abrogation of infection (on detection of CAA) will be preferable to 12-week “admissions”; but views from the communities will influence our future approach.

Controlled human infections with known pathogens inevitably involve risks and possibly the burden of symptoms. Available mitigations in several examples reduced our risk scores only to moderate, rather than low: for example, symptomatic treatment and early abrogation of infection cannot reduce the likelihood of symptoms below common, but can reduce the impact of the symptoms. Such areas emphasise the need for caution – for example, small group sizes and carefully monitored dose-escalation approaches.

We realize that symptoms may be different among Ugandan volunteers than among Dutch volunteers. Particularly, Katayama fever is considered less likely to occur in subjects from endemic, compared to subjects from non-endemic settings ^1^. Nevertheless, we shall provide full information to potential volunteers about symptoms predicted from the literature, and those which occurred previously in the Dutch volunteers. We are currently piloting educational materials, volunteer information sheets, and tests of comprehension in order to ensure that Ugandan volunteers can be enrolled with genuine understanding and fully informed consent. As well, we shall work with community leaders and advisors to ensure optimal understanding of the work, and to mitigate the impact of rumours about the work which are likely to arise.

We conclude that, with careful risk management, CHI-S can be safely implemented in Uganda with a view to accelerating vaccine development against this important communicable disease.

## DisclaimerData availability

### Underlying data

No data are associated with this article.

### Extended data

Open Science Framework: Controlled Human Infection Model – Schistosomiasis. https://doi.org/10.17605/OSF.IO/53GT9 ^9^

This project contains the following extended data:

- Appendix 1.docx (risk assessment report addressing the intended transfer to and culturing of the snail *Biomphalaria glabrata* in Uganda)
- Appendix 2.docx (Summary of safety precautions for working with Schistosoma)

Data are available under the terms of the Creative Commons Zero "No rights reserved" data waiver (CC0 1.0 Public domain dedication).
