# Supplementary material for: Risk assessment for the implementation of controlled human Schistosoma mansoni infection trials in Uganda
Source: AAS Open Res. 2019 Aug 13;2:17. Originally published 2019 Jun 3. [Version 2] doi: 10.12688/aasopenres.12972.2 (PMC6901351; doi:10.12688/aasopenres.12972.2)
Supplement: Peer review history

# Peer review: Reviewer response for version 2

**DOI:** 10.21956/aasopenres.14072.r27120

**Published:** 2019-08-21

**Authors:** Donald Harn

**Affiliations:** 1 Department of Infectious Diseases, College of Veterinary Medicine, Center for Tropical and Emerging Global Diseases, University of Georgia, Athens, GA, USA

I confirm that I have read this submission and believe that I have an appropriate level of expertise to confirm that it is of an acceptable scientific standard.

# Peer review: Reviewer response for version 1

**DOI:** 10.21956/aasopenres.14053.r26958

**Published:** 2019-06-26

**Authors:** Donald Harn

**Affiliations:** 1 Department of Infectious Diseases, College of Veterinary Medicine, Center for Tropical and Emerging Global Diseases, University of Georgia, Athens, GA, USA

Overall, the authors have put together 3 different scenarios that would eventually provide for controlled human infections with male schistosomes in Uganda. The authors present good rationale for why such CHI may be valuable for vaccine trials, and I generally agree with their rationale. 

 The authors produced a document that attempts to summarize the risks associated with each scenario and how they would be reduced if proper interventions were imposed. Overall, a nicely written paper that concludes that “Option 2” introducing the Puerto Rican strain of *Schistosoma mansoni*into Ugandan labs, and possibly the ecology, is the best way forward. I disagree with this assessment as outlined here and feel that the more costly (initially) “Option 3” is the best way forward to minimize unknown potential ecological risks. 

 A few things concern me. One, the authors state “The level of risk and effectiveness of proposed controls was determined by consensus between the authors.” Some of these authors were involved in establishing CHI in Dutch volunteers in Leiden. However, I am concerned with the level of expertise the authors have in ecology or ecologic modeling to accurately assess the likelihood of introduction of new parasite or snail into the environment, and the impact of such new species into the Ugandan environment?

 For Option 3 the authors state“There are however several challenges with using Ugandan snails and isolates. With regard to Ugandan snail species, there is variability between snail species in susceptibility to S. mansoni infection;”  Wouldn’t this concern be the same for option 2.?

 Also under Option 3, the authors state “With regard to the new schistosome laboratory strain, the characteristics of this would be unknown in terms of virulence and susceptibility to praziquantel treatment. Determining these characteristics would not be simple, since validated tests for schistosome resistance are currently not available. In addition, the new isolate would not be clonal and variability within the newly collected schistosome population might result in variable responses in the host, and to drug treatment. In addition, dose-finding studies would start from scratch to find the balance between tolerability and attack rate.”

 I find all of these arguments not justifiable. Determining susceptibility to PZQ in their animal model is straightforward and they can do this. They can easily produce a clone or clones of schistosomes to initiate these studies. Yes, infectious dose studies will have to start from scratch, but in reality, as this is an endemic population, not Dutch volunteers, this will have to be done with Ugandans anyway.

 In the “Natural Infection during trial period” the authors note that at some point they may introduce female cercariae infections. Are they implying single-sex female cercariae infections? Later in this section the authors note that infected individuals should avoid any contact with schistosome contaminated water. How feasible is this? Much of the risk here will depend on the residence of the cohorts for the Ugandan CHI trials. If the volunteers are urban, with little to no chance of encountering contaminated water, this point is moot and perhaps the authors have considered this as a likely way to mitigate this potential problem.

 General, the Tables are informative. I may have missed this but, it is not clear to me how the authors determined the reduced “Total risk post control” score? We can see large swings in score from initial “Total inherent risk” to the score for “Total risk post control” but do not have a numerical rationale mentioned or discussed for why this lower score.

 Minor:

 Potent should be patent

 Hybridisation with local schistosome population unlikely to result in praziquantel resistance? Maybe true, but you still have created a hybrid parasite.

 Under “Option 1: snail culture facilities, potential ecological harm” page 5, first paragraph, line 4 should be corrected to read “access to the laboratory should **be** controlled…”

I confirm that I have read this submission and believe that I have an appropriate level of expertise to confirm that it is of an acceptable scientific standard, however I have significant reservations, as outlined above.

# Response

**Published:** 2019-08-05

**Authors:** Alison Elliott

**Affiliations:** Uganda Virus Research Institute, Uganda

**We thank Dr Harn for his review.  Our response is as follows.**

 Comment. The authors produced a document that attempts to summarize the risks associated with each scenario and how they would be reduced if proper interventions were imposed. Overall, a nicely written paper that concludes that “Option 2” introducing the Puerto Rican strain of Schistosoma mansoni into Ugandan labs, and possibly the ecology, is the best way forward. I disagree with this assessment as outlined here and feel that the more costly (initially) “Option 3” is the best way forward to minimize unknown potential ecological risks.

 Response. We agree that the choice between the safety concerns on the one hand and the ecological risk on the other, is a challenging one. We also understand from this comment that we have not been clear about why, on balance, we prefer option 2. We have extended the paragraph in the discussion on this decision to be more specific about why this is our current preference.

 Comment. A few things concern me. One, the authors state “The level of risk and effectiveness of proposed controls was determined by consensus between the authors.” Some of these authors were involved in establishing CHI in Dutch volunteers in Leiden. However, I am concerned with the level of expertise the authors have in ecology or ecologic modeling to accurately assess the likelihood of introduction of new parasite or snail into the environment, and the impact of such new species into the Ugandan environment?

 Response. We agree that we, the authors, have little experience in assessment of ecological risks. For these parts of the risk assessment, we therefore consulted relevant ecologists and geneticists including Dr A. J. de Winter (Naturalis Biodiversity Center, Leiden, The Netherlands) regarding the snails and Dr. M. Berriman (Wellcome Trust Sanger Institute, UK) regarding the parasite. To make this clear, we have better outlined the considerations. With his permission, we have added Dr. Berriman to the acknowledgements.

 Comment. For Option 3 the authors state “There are however several challenges with using Ugandan snails and isolates. With regard to Ugandan snail species, there is variability between snail species in susceptibility to S. mansoni infection;”  Wouldn’t this concern be the same for option 2.?

 Response. This is correct. We have adjusted accordingly and mention this concern for option 2 as well.

 Comment. Also under Option 3, the authors state “With regard to the new schistosome laboratory strain, the characteristics of this would be unknown in terms of virulence and susceptibility to praziquantel treatment. Determining these characteristics would not be simple, since validated tests for schistosome resistance are currently not available. In addition, the new isolate would not be clonal and variability within the newly collected schistosome population might result in variable responses in the host, and to drug treatment. In addition, dose-finding studies would start from scratch to find the balance between tolerability and attack rate.”

 I find all of these arguments not justifiable. Determining susceptibility to PZQ in their animal model is straightforward and they can do this. They can easily produce a clone or clones of schistosomes to initiate these studies. Yes, infectious dose studies will have to start from scratch, but in reality, as this is an endemic population, not Dutch volunteers, this will have to be done with Ugandans anyway.

 Response. We agree that an inbred population of Ugandan schistosomes could be established in a rodent model, but are not confident that it would be straightforward. Many generations of crossing following initial infection with a clone of males and a clone of females would be required to produce a monomorphic strain. Initiating the strain with a single clone of males and a single clone of females would minimise variability, but might also result in quite atypical parasites, not necessarily representative of the Ugandan population of schistosomes in general.  Starting with a more diverse selection of cercariae would generate a more representative laboratory population of Ugandan schistosomes, but would mean that the characteristics of any particular clone (notably pathogenicity or praziquantel resistance) selected for CHI-S would be unpredictable.  

 Indeed our greatest concern is the potential for praziquantel resistance. Ugandan populations have been exposed to regular praziquantel treatment for over a decade, so there is a risk that the initial isolates would include individuals with relative praziquantel resistance ^1^  which could not be established with certainty in the initial stages of the above process.  While we agree that some testing could be done in animals the pharmacokinetics and pharmacodynamics of praziquantel are complex and different between rodents and humans. ^2,3^

 With regards to the dose-finding study, we agree that this would be the case for any of the three options and therefore removed this sentence.

 Comment. In the “Natural Infection during trial period” the authors note that at some point they may introduce female cercariae infections. Are they implying single-sex female cercariae infections? Later in this section the authors note that infected individuals should avoid any contact with schistosome contaminated water. How feasible is this? Much of the risk here will depend on the residence of the cohorts for the Ugandan CHI trials. If the volunteers are urban, with little to no chance of encountering contaminated water, this point is moot and perhaps the authors have considered this as a likely way to mitigate this potential problem.

 Response. We have clarified the sentence on female cercariae – we indeed plan on developing a single-sex female model in the future.

 With regard to avoiding contact with contaminated water, the location we are considering to recruit volunteers for this work is a peri-urban fishing village close to Entebbe, well known to the research team, where many communal taps are available to provide an alternative water source. In addition, it will be possible to select volunteers who have access to adequate sanitation. We thus envision that it will be feasible to avoid any lake contact for these inhabitants. Through surveys and group discussions, we are currently assessing possible strategies to incentivise the use of tap water – for example, making it freely available for study participants. We hope that this will allow participation from the true target population i.e. people with previous exposure to *Schistosoma mansoni*.

 Comment. General, the Tables are informative. I may have missed this but, it is not clear to me how the authors determined the reduced “Total risk post control” score? We can see large swings in score from initial “Total inherent risk” to the score for “Total risk post control” but do not have a numerical rationale mentioned or discussed for why this lower score.

 Response. The inherent risk was defined as the risk before putting controls in place, calculated as the product of the likelihood and impact scores. The residual risk was similarly calculated, based on likelihood and impact scores after controls have been put in place. The difference in the calculated scores is based on the degree to which the proposed controls are expected to alter either the likelihood of the event happening, or the impact of the event, should it happen. We have added the scores to the legend of the tables for clarity.

 Comment. Potent should be patent

 Response. We have changed this.

 Comment. Hybridisation with local schistosome population unlikely to result in praziquantel resistance? Maybe true, but you still have created a hybrid parasite.

 Response. This is true. We have added a sentence to include this concern.

 Comment. Under “Option 1: snail culture facilities, potential ecological harm” page 5, first paragraph, line 4 should be corrected to read “access to the laboratory should be controlled…”

 Response. We have corrected this.

**References**

 1.         Crellen T, Walker M, Lamberton PH, Kabatereine NB, Tukahebwa EM, Cotton JA, et al. Reduced Efficacy of Praziquantel Against Schistosoma mansoni Is Associated With Multiple Rounds of Mass Drug Administration. Clin Infect Dis. 2016 Nov 1;63(9):1151-9

 2.         Abla N, Keiser J, Vargas M, Reimers N, Haas H, Spangenberg T. Evaluation of the pharmacokinetic-pharmacodynamic relationship of praziquantel in the Schistosoma mansoni mouse model. PLoS Negl Trop Dis. 2017 Sep;11(9):e0005942

 3.         Bustinduy AL, Waterhouse D, de Sousa-Figueiredo JC, Roberts SA, Atuhaire A, Van Dam GJ, et al. Population Pharmacokinetics and Pharmacodynamics of Praziquantel in Ugandan Children with Intestinal Schistosomiasis: Higher Dosages Are Required for Maximal Efficacy. MBio. 2016 Aug 9;7(4)

# Peer review: Reviewer response for version 1

**DOI:** 10.21956/aasopenres.14053.r27005

**Published:** 2019-06-17

**Authors:** James E. Meiring

**Affiliations:** 1 Malawi-Liverpool Wellcome Trust Clinical Research Programme, Blantyre, Malawi; 2 Oxford Vaccine Group, Oxford University, Oxford, UK

This is a well written, and referenced letter outlining the risk assessments for setting up CHI-S in Uganda. The authors should be commended for a very thorough approach to the topic, highlighting all areas of risk, from shipment and storage of infectious diseases and animal hosts through to the community-based responses to CHI-S and possible impact on other research activity.

 In addition to what is presented here, I think it might be helpful for the authors to outline their thinking on expectations for financial reimbursement for participants who go through the trial in Uganda, and if there are any risks attached to this, as discussed in Gordon, S et al. 2018 published in Wellcome Open Research ^1^.

 It may also be beneficial to consult the literature for other examples of CHI studies that have been moved from non-endemic, high income countries to endemic sites. Although the individual risks may be different, the general lessons may be applicable and helpful.

I confirm that I have read this submission and believe that I have an appropriate level of expertise to confirm that it is of an acceptable scientific standard.

## References

[1] : A Malawi guideline for research study participant remuneration. *Wellcome Open Res*.2018;3: 10.12688/wellcomeopenres.14668.2 141 10.12688/wellcomeopenres.14668.2 30662959PMC6329041

# Response

**Published:** 2019-08-05

**Authors:** Alison Elliott

**Affiliations:** Uganda Virus Research Institute, Uganda

Comment: In addition to what is presented here, I think it might be helpful for the authors to outline their thinking on expectations for financial reimbursement for participants who go through the trial in Uganda, and if there are any risks attached to this, as discussed in Gordon, S et al. 2018 published in Wellcome Open Research.

Response: We agree that the financial reimbursement is an important topic for debate. We have thus added a paragraph on this matter and included it in the table with risks related to the controlled human infection with *Sm.*

Comment: It may also be beneficial to consult the literature for other examples of CHI studies that have been moved from non-endemic, high income countries to endemic sites. Although the individual risks may be different, the general lessons may be applicable and helpful.

Response: We agree that it will be essential to interact with experienced researchers who have implemented CHI studies in LMIC. We added reference to this important topic by addressing a paper on the first malaria CHI in Kenya that describes issues that are also applicable for our CHI-S.
